# Evaluation of potential helium source rocks and helium enrichment factors in coalbed methane in the eastern margin of Ordos basin

**DOI:** 10.1038/s41598-025-28531-3

**Published:** 2026-01-13

**Authors:** Yue Chen, Shizhen Tao, Rui Kang, Jianrong Gao, Yiqing Yang, Xiang Fang, Wei Song, Yanyan Chen, Xiangbai Liu

**Affiliations:** 1 PetroChina Research Institute of Petroleum Exploration and Development, Beijing, 100083 China; 2 Research Institute of Exploration and Development, PetroChina Changqing Oilfield Company, Xi’an, 710018 China

**Keywords:** Helium, Coalbed methane, Helium source rocks, Helium production rate, Ordos basin, Energy science and technology, Solid Earth sciences

## Abstract

As a non-renewable noble gas with important industrial value, helium has significant implications for its resource exploration and development. The Ordos basin has become a core area for research on the generation, release, and enrichment laws of helium due to its unique geological structure, abundant stratigraphic lithology, and complex sources of helium in coalbed methane. We conducted analyses of gas compositions for coalbed methane samples and determined the U and Th contents for rock samples from the eastern margin of the Ordos basin. Based on these results, we calculated the helium generation potential of helium source rocks of different stratigraphic ages and lithologies in the same region. The results show that the helium content of 8 wells in the north of Sanjiao area is higher than 0.05%, and the helium content of 4 wells is greater than 0.1%, which meets the industrial helium extraction standard. The U and Th content and the volume of ^4^He released per gram of rock per year in different geological ages showed that the Carboniferous > Proterozoic > Permian > Archean > Ordovician > Cambrian, and the volume of ^4^He generated by radioactive decay per cubic meter of rock: Proterozoic > Archean > Carboniferous > Permian > Ordovician > Cambrian. In terms of different lithologies, the contents of U and Th and the volume of ^4^He released per gram of rock per year showed bauxite rock > coal > mudstone > basement rock> sandstone > carbonate rock, and the volume of ^4^He produced by the radioactive decay of each cubic meter of rock is: basement rock> bauxite rock > mudstone > coal > sandstone > carbonate rock. In summary, although the Carboniferous and bauxite rocks have high contents of U and Th and helium generation rates, the Archean-Proterozoic basement has the characteristics of ancient rock age and large development scale, and a large amount of ^4^He will accumulate during the radioactive decay of rocks. This fully indicates that the stratigraphic age, scale, and distribution, etc., play a key role in the helium generation process and will have a profound impact on the distribution pattern of regional helium resources, providing an important basis for in-depth exploration of the helium reservoir formation mechanism and subsequent resource exploration and development.

## Introduction

Helium, as a noble gas that plays a crucial role in the field of modern science and technology, has shown a wide range of applications in cutting-edge industries such as aerospace, medical and electronics industries. It has the characteristics of extremely low melting, boiling points, chemical reactivity and solubility in water. Moreover, due to its tiny molecular diameter, it shows extremely strong diffusion properties. Currently, it is known that there are three main sources of helium: one is atmospheric helium; The second is radiogenic helium, which is closely related to the α decay process of radionuclides such as ^238^U, ^235^U, and ^232^Th in the earth’s crust; And the third is mantle-derived helium, which has an inherent connection with the activities and evolution of deep mantle materials within the Earth^1^. In the cosmic space, the abundance of helium is significantly high. However, in the Earth’s material system, the abundance of helium is extremely low, and it is diffusely distributed in various material forms in nature. As the global high-tech industries burgeon and thrive rapidly, the market requirement for helium is showing a sharp growth trend.

The Ordos basin is the most tectonically stable petroliferous basin. It’s also the largest natural gas production area and the most abundant coalbed methane resources in China^2^. The coalbed methane resources in this basin are mainly concentrated in the eastern margin of the Ordos basin. At present, the presence of helium has been detected in several gas fields in the Ordos basin. For example, the average helium content in Dongsheng gas field is 0.133%^3^, The helium content within the Sulige Gas Field lies in the interval of 0.02% to 0.18%, with an average value of 0.05%. Meanwhile, in the Qingyang gas field, the helium content spans from 0.121% to 0.204%^4^, and its average is 0.144%. In coalbed methane, although helium is also detected, the content is low in most areas and it is difficult to reach the industrial development value. The survey results show that the helium content in the coalbed methane is between 0.000052% and 0.003325%, with an average value of 0.00086% in the Qinshui basin^5^, and the helium content in the Liupanshui basin ranges from 0.0041% to 0.1136% and the average value is 0.0336%^6^. The helium content in Hancheng area of the eastern margin of the Ordos basin is between 0.0001% and 0.0867%, the helium content in Daning-Jixian is between 0.0025 and 0.0421%. In the northern part of the Sanjiao area, there is a relatively high helium content, which ranges from 0.0379 to 0.2019% and its average is 0.0930%^7^. Coalbed methane is a typical self-generated and self-reservoired gas reservoir. Previous studies suggest that high hydrocarbon generation intensity dilutes helium, making it difficult for coal-measure gas reservoirs to form helium-rich gas reservoirs. However, the discovery of gas reservoirs with high helium content in the north of Sanjiao area indicates that coal-measure gas reservoirs may also form helium-rich gas reservoirs under specific conditions. In addition, through the calculation of in-situ autogenous helium, previous researchers found that the measured helium content in coal-measure natural gas at the eastern margin of the Ordos basin is higher than the in-situ autogenous helium content, which indicates the presence of exogenous helium supplementation^7^. Therefore, this study selected the north of Sanjiao area in the eastern margin of the Ordos basin as the key research object, collected natural gas samples from 10 coalbed methane wells in the study area, and carried out component testing and analysis, so as to lay a data foundation for in-depth exploration of the related characteristics of helium in coalbed methane.

The Ordos basin exhibits the remarkable characteristics of multi-layered and multiple sets of source rocks. All types of rocks can generate trace amounts of helium and continuously produce helium. In general, ancient rocks with high U and Th content are considered to be effective helium source rocks. Rocks such as granite have been identified as potential helium source rocks^8^, and bauxite rock and black shale also have high U and Th content^9,10^. On this basis, 59 helium source rock samples were collected from the Carboniferous-Permian, Ordovician, Cambrian, and Proterozoic-Archean basement (lithology includes basement samples (such as granite, gneiss, diorite, granite gneiss, metamorphic sandstone, etc.), as well as bauxite rock, coal, mudstone, sandstone, and carbonate rock, and the trace element content test was carried out, and the trace element data of 18 basement rock samples from the eastern margin of the Ordos basin were collected from the Exploration and Development Research Institute of Changqing Oilfield. The samples were classified according to different geological ages and lithologies. Considering the geological background, parameters such as the rock ages, densities and thicknesses of different geological ages and lithologies in the study area were investigated. We computed the volume of ^4^He released per gram of rock annually and the amount of ^4^He produced through radioactive decay reactions in each cubic meter of rock. The aim is to clarify the material source and formation mechanism of helium in the coal-measure natural gas of the Ordos basin and to find out the effective potential types of helium source rocks in the study area.

## Geological setting

As a secondary structural unit of the North China Platform, the Ordos basin is a large petroliferous basin developed on the craton basement, and its natural gas resource potential is extremely considerable^11^. The aggregate area of the basin is 37 × 10^4^ km^2^, which is segmented into six first-order structural units: the western margin thrusting belt, Tianhuan depression, Weibei uplift, Yishan slope, Yimeng uplift and Jinxi flexure belt. During the long geological history, the Ordos basin has undergone multiple tectonic cycles such as Lüliang, Jinning, Caledonian, Hercynian, Indochina, Yanshan, and Himalayas movements. Among them, the Caledonian movement caused the basin to be uplifted and denuded, which led to the loss of Silurian and Devonian in the basin as a whole, and only the Benxi Formation remaining in the Carboniferous^12^.

**Fig. 1:**
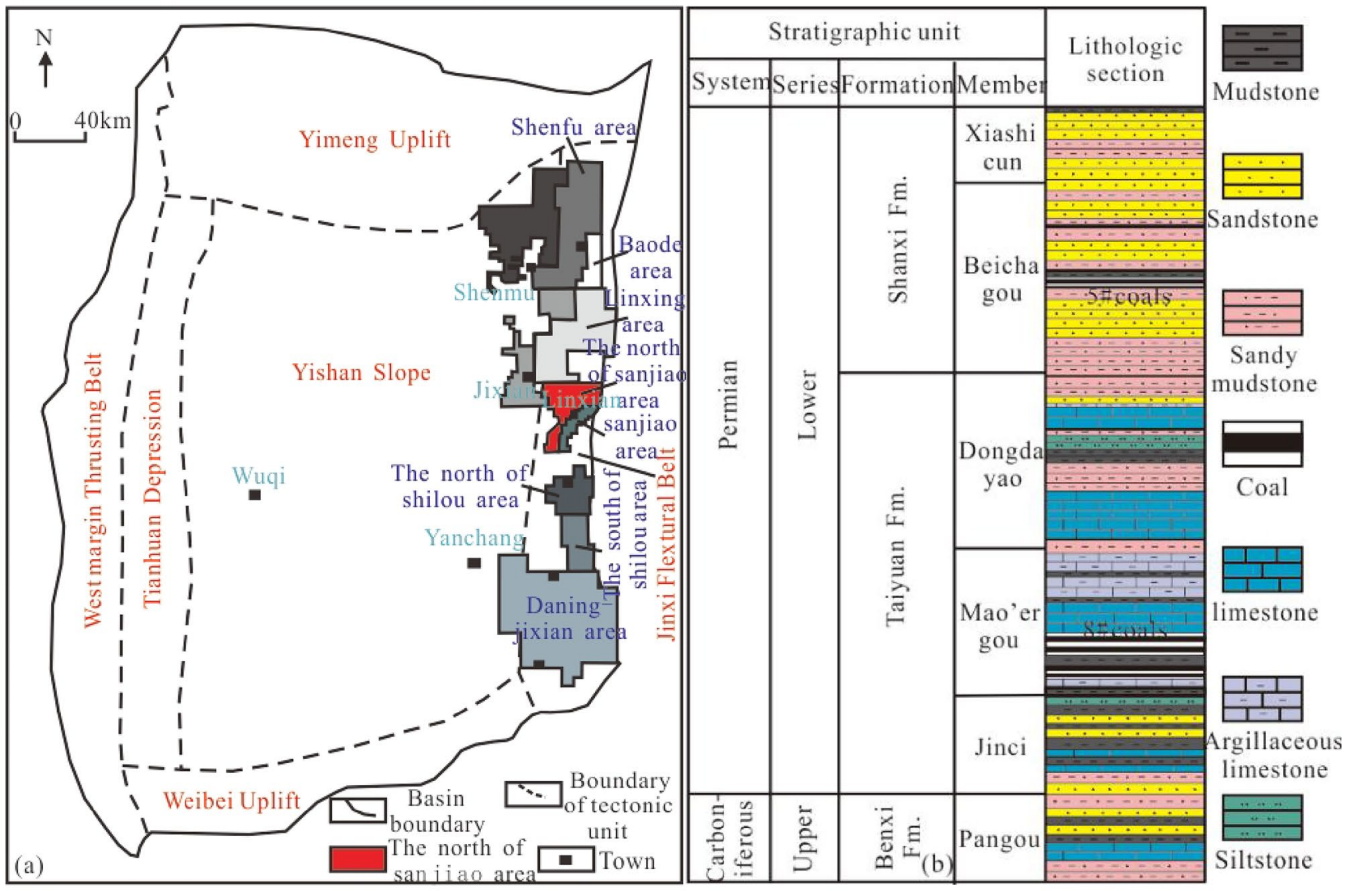
Tectonic units, location of the study area (**a**) and comprehensive stratigraphic column (**b**) of the Ordos basin. Modified from refs.^14,16^.

The coalbed methane resources are mainly concentrated in the Jinxi flexure belt on the eastern margin of Ordos basin. Jinxi flexure belt is approximately 400 km long from north to south and 50 km wide from east to west. It is closely adjacent to the Yellow River on the western side, and is separated from the Lüliang Uplift by the Lishi Fault on the eastern side. It starts from Jungar Banner in the north and extends to Hejin-Hancheng in the south. This flexure belt presents an overall situation of tilting eastward and plunging westward in terms of terrain, and it belongs to a large monocline structure. Its strata are relatively complete in development and have a stable “dual structure”, which is formed of the lower metamorphic basement and the upper sedimentary cover. Compared with other areas within the basin, Jinxi flexure belt is in an uplifted form, and the sedimentary cover has a relatively small thickness^13^. The north of Sanjiao coalbed methane block is situated in the middle between Jinxi flexure belt and the Hedong coalfield geological area in Shanxi Province, with an area of 282 km^2^ ^14^. In this area, the key coal-bearing strata are the Lower Permian Taiyuan Formation as well as the Shanxi Formation (Fig. 1)^15^.

### Samples and analytical methods

A total of 59 field outcrop samples were collected from the eastern margin of the Ordos basin in this study. The sampling work focused on the Liulin and Baode areas, covering multiple sets of formations: these include basement rocks of the Archaean-Proterozoic (Archaean samples are from the Jiehekou Group and Lüliang Group, Proterozoic samples from the Yejishan Group and Lanhe Group, with lithologies including granite, gneiss, schist, granitic gneiss, etc.), as well as rocks from the Shanxi Formation, Taiyuan Formation of the Permian System and Benxi Formation of the Carboniferous System. Samples of the Cambrian System and Ordovician System were mainly collected from the Liulin area in the eastern margin of the basin. The trace element test and analysis of all rock samples were completed at Tianjin University. In this work, an inductively coupled plasma-mass spectrometer (ICP-MS) was used to determine the concentrations of trace elements such as U and Th in the samples. The relative standard deviation of the sample determination results is less than 5%. 10 natural gas samples were gathered from the northern part of the Sanjiao area located on the eastern margin of Ordos basin and tested natural gas components in the China National Petroleum Corporation Natural Gas Formation and Development Key Laboratory. The analysis method can be found in Wang et al.^17^.

## Results

### Helium content

The results of coalbed methane samples from 10 wells in the north of Sanjiao area showed that the natural gas component was mainly CH_4_, and the CH_4_ content was between 89.22 and 97.03%. The N_2_ content ranges from 0.13 to 2.27%; The CO_2_ content is very low, ranging from 0 to 2.31%; The He content is between 0.02 and 0.16%, with an average is 0.082%. Among them, the helium content in 4 wells is greater than 0.1%, and the helium content in 8 wells is greater than 0.05% (Table 1).

**Table 1:** Coalbed methane natural gas components in Sanjiao North area.

Well	System	He/%	H_2_/%	*N*_2_/%	CO_2_/%	H_2_S/%	C_1_/%	C_2_/%	C_3_/%	iC_4_/%	nC_4_/%	iC_5_/%	nC_5_/%	C_6+_/%
SJB15	P	0.06	0.04	0.47	0.00	0.00	89.55	7.36	1.71	0.27	0.29	0.10	0.06	0.10
SJB16	P	0.09	0.00	0.83	0.00	0.00	93.36	4.08	1.00	0.22	0.21	0.09	0.05	0.07
QJS Gas gathering station	P	0.09	0.01	1.50	0.34	0.00	90.75	5.20	1.34	0.25	0.27	0.10	0.06	0.08
SJB18	P	0.05	0.03	0.73	0.87	0.00	89.22	6.58	1.67	0.27	0.33	0.12	0.07	0.07
SJB32	P	0.11	0.01	1.29	0.00	0.32	97.03	0.98	0.16	0.04	0.04	0.02	0.01	0.00
SJB19	P	0.10	0.01	2.27	0.00	0.00	92.99	3.39	0.79	0.15	0.16	0.06	0.04	0.05
TB-02	P	0.03	0.00	0.70	0.30	0.00	90.30	6.74	1.33	0.23	0.19	0.08	0.04	0.06
SJB1-10	P	0.02	0.02	0.13	2.31	0.00	93.01	3.46	0.70	0.14	0.13	0.05	0.03	0.00
SJB23	P	0.11	0.01	1.70	0.73	0.00	93.84	2.84	0.51	0.10	0.09	0.04	0.02	0.00
SJB-1	P	0.16	0.00	2.09	0.00	0.00	94.57	2.46	0.47	0.10	0.09	0.04	0.02	0.00

### Helium source rocks

There are two main isotopes of helium on the Earth, namely ^3^He and ^4^He. Among them, ^3^He mainly derives from the degassing of the deep mantle, while ^4^He is produced by the radioactive decay of rocks that contain a high concentration in U and Th elements. Usually, the ^3^He/^4^He isotopic characteristics are used to identify the source of helium. Related studies demonstrate that the source of the Ordos basin’s helium resources is essentially crustal origin^18–20^. Taking the north of Sanjiao area as an example, the content of mantle-derived He is extremely low, and the percentage of helium originating from the mantle is below 1%^7^. From this, it can be known that the helium gas in the study area is mainly^4^He generated by the radioactive decay of rocks rich in U and Th elements.

**Fig. 2:**
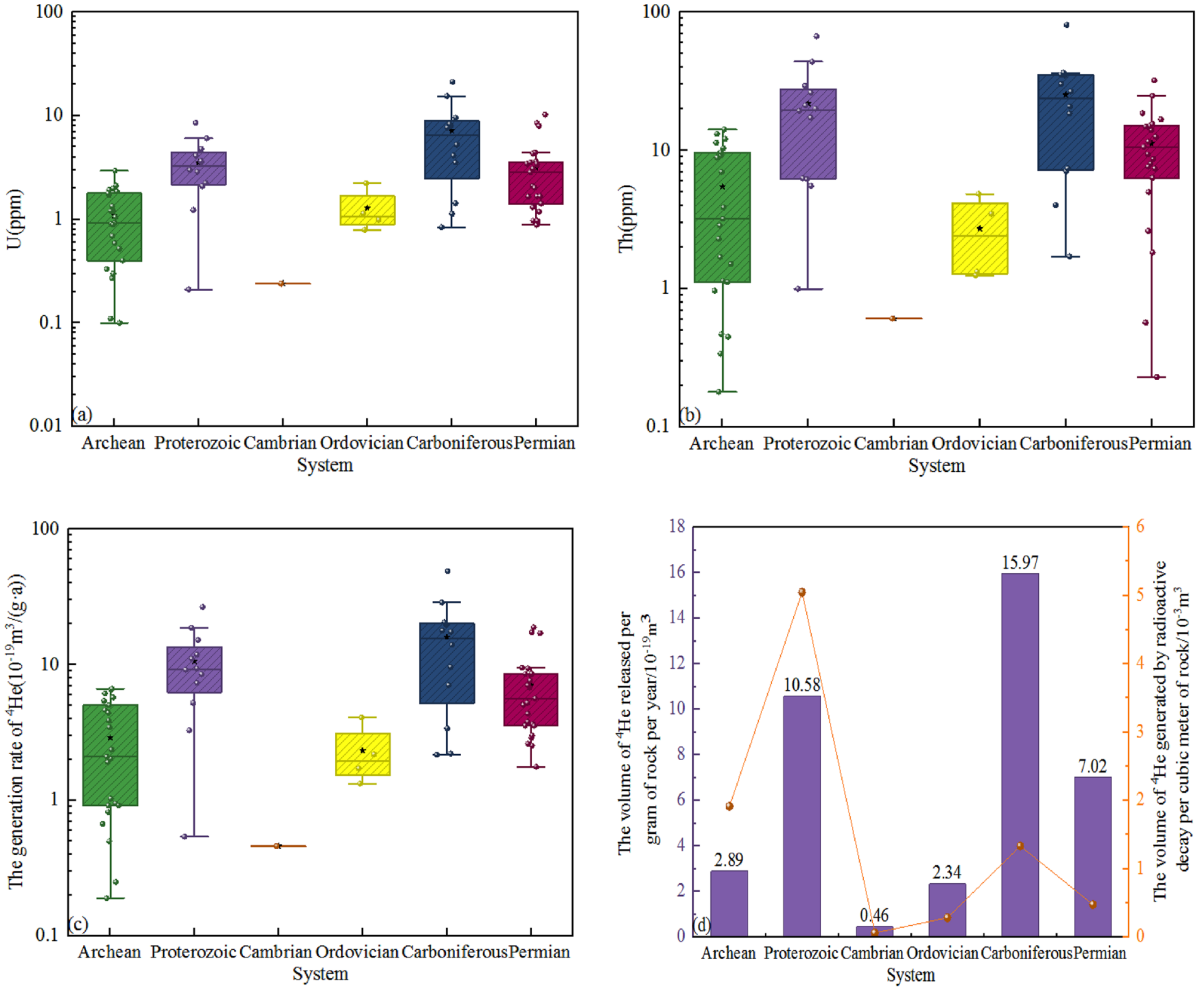
Relationship diagrams between rocks of different geological ages and U (**a**), Th (**b**), helium generation rate (**c**), and helium release potential (**d**).

As radioactive elements, U and Th directly determine the helium-generation rate of rock formations. The higher their contents, the greater the number of atoms undergoing decay per unit time, and the larger the amount of ^4^He produced, thus affecting the helium-generation rate of rock formations. Trace element analysis was carried out on rock samples from different geological ages in the study area (the Permian, Carboniferous, Ordovician, Cambrian, Proterozoic, and Archean). The results are as follows: 12 samples from the Carboniferous System have the highest U and Th contents, with average values reaching 7.21 ppm and 25.35 ppm, respectively. This is followed by 12 samples from the Proterozoic, which have average U and Th contents of 3.53 ppm and 21.93 ppm, respectively. Samples from the Permian System, Archaean, and Ordovician System have relatively lower U and Th contents. The Cambrian System samples have the lowest U and Th contents, both of which are less than 1 ppm. In terms of the contents of U and Th, the order is Carboniferous > Proterozoic > Permian > Archean > Ordovician > Cambrian (Table 2; Fig. 2a, b).

**Fig. 3:**
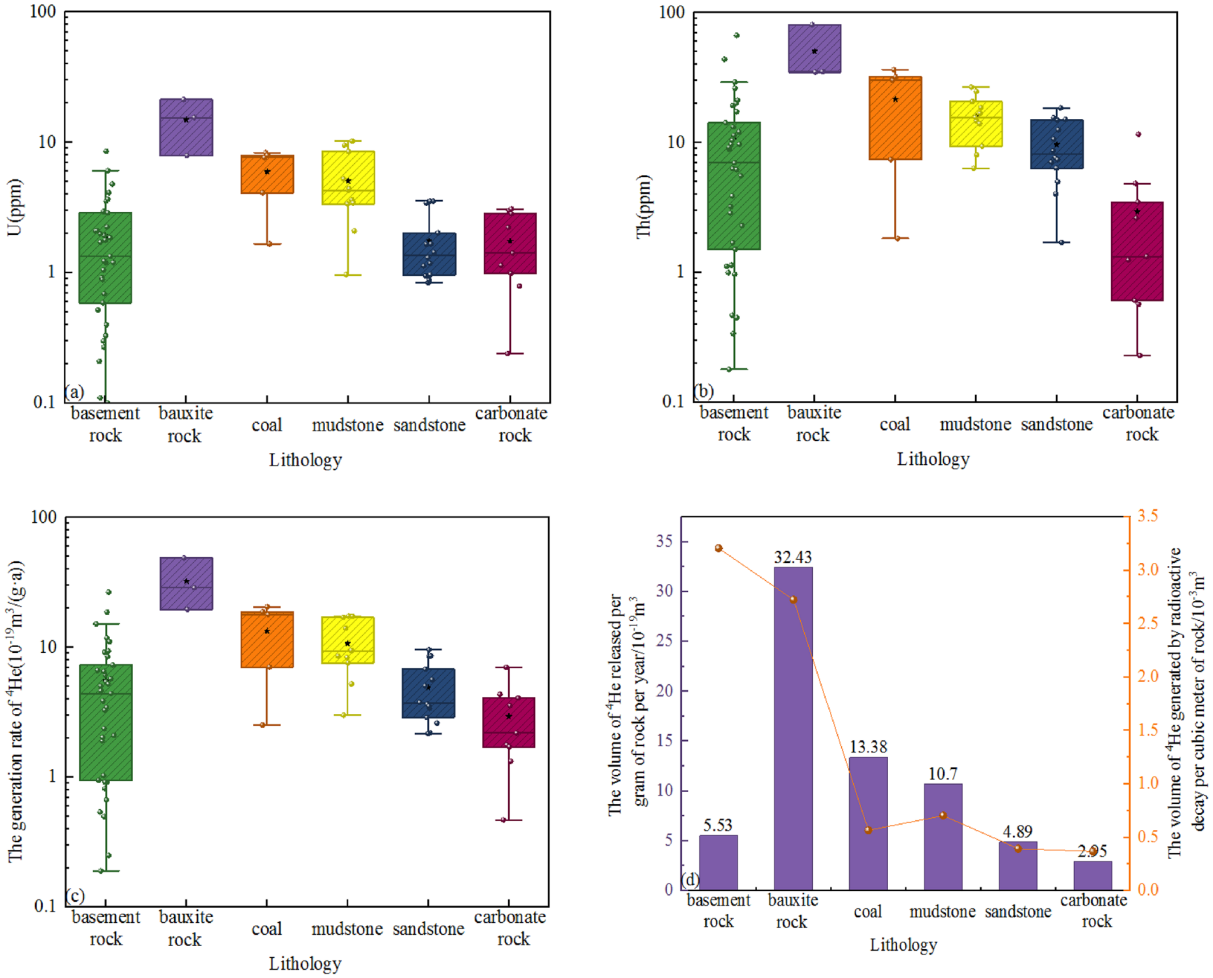
Diagrams of the relationships between different lithologies and U (**a**), Th (**b**), helium generation rate (**c**), and helium release potential (**d**).

Analyses of various rock samples, including basement rock, bauxite rock, coal, mudstone, sandstone, and carbonate rock, indicate that the bauxite rock in the Benxi Formation contains high U and Th. The highest content of U can reach 21.29 ppm, and the content of Th is as high as 80.97 ppm. The outcrop samples of five coal rocks from Shanxi Formation and Benxi Formation also have high U and Th contents. The content of U is 1.67 to 8.34 ppm, and the content of Th is 1.83 to 36.47 ppm, with average contents of 5.95 ppm and 21.61 ppm, respectively (Table 3). In terms of the contents of U and Th, the order is bauxite rock > coal > mudstone > basement rock > sandstone > carbonate rock (Fig. 3a, b). The contents of U and Th in the Proterozoic-Archean basement rock fluctuate greatly, among which the contents of U and Th in granite are higher than those in granitic gneiss and gneiss. These rocks abundant in U and Th provide an abundant material source for the helium gas in the coalbed methane in the eastern margin of the Ordos basin.

**Table 2:** The contents of U and Th in rocks of different geological ages.

System	Number of samples	U(ppm)	Th(ppm)
Permian	25	$\frac{{0.88\sim 10.24}}{{3.14}}$	$\frac{{0.23\sim 32.10}}{{11.25}}$
Carboniferous	12	$\frac{{0.84\sim 21.29}}{{7.21}}$	$\frac{{1.71\sim 80.97}}{{25.35}}$
Ordovician	4	$\frac{{0.79\sim 2.23}}{{1.29}}$	$\frac{{1.25\sim 4.86}}{{2.74}}$
Cambrian	1	0.24	0.61
Proterozoic	12	$\frac{{0.21\sim 8.55}}{{3.53}}$	$\frac{{1.00\sim 67.05}}{{21.93}}$
Archean	23	$\frac{{0.10\sim 2.95}}{{1.09}}$	$\frac{{0.18\sim 14.21}}{{5.47}}$

**Table 3:** U and Th contents of different types of rocks.

Lithology	Formation	Number of samples	U(ppm)	Th(ppm)
Basement rock	Pt-Ar	35	$\frac{{0.10\sim 8.55}}{{1.93}}$	$\frac{{0.18\sim 67.05}}{{11.12}}$
Bauxite rock	C_2_b	3	$\frac{{7.93\sim 21.29}}{{14.97}}$	$\frac{{34.94\sim 80.97}}{{50.41}}$
Coal	C_2_b, P_1_s	5	$\frac{{1.67\sim 8.34}}{{5.95}}$	$\frac{{1.83\sim 36.47}}{{21.61}}$
Mudstone	C_2_b, P_1_s, P_1_t	11	$\frac{{0.97\sim 10.24}}{{5.07}}$	$\frac{{6.36\sim 26.73}}{{16.00}}$
Sandstone	C_2_b, P_1_s, P_1_t, P_2_h	14	$\frac{{0.84\sim 3.55}}{{1.75}}$	$\frac{{1.71\sim 18.51}}{{9.66}}$
Carbonate rock	P_1_t, O_1_m, O_1_y, Є_3_f	9	$\frac{{0.24\sim 3.09}}{{1.75}}$	$\frac{{0.23\sim 11.60}}{{2.95}}$

Compared with the Hancheng area and the Daning-Jixian area, the helium content in the north of the Sanjiao area is higher, which may be attributed to the presence of the Zijinshan Rock Mass. The Zijinshan Rock Mass is located about 20 km northwest of Linxian, Shanxi Province, and its outcrop area reaches 23 km^2^^21^. The average contents of U and T elements in this rock mass are 2.595 ppm and 7.085 ppm, respectively^22^. Based on the previous research results, the Zijinshan Rock Mass was formed during the time period from 138.7 to 125.0 Ma, and it is a product of the Early Cretaceous alkaline magmatic event^23^.

The Zijinshan alkaline complex was formed in an extensional environment after the Mesozoic tectonic regime transition in the North China Craton. During the process of tectonic regime transformation, the powerful stress caused the asthenosphere in the Lüliang area to surge upward multiple times. Subsequently, the magma generated by the partial melting of the asthenosphere, the lithospheric mantle, and the lower crustal materials underwent multiple mixing processes in different proportions. These mixed magmas of various stages successively intruded upward in batches and were emplaced in the shallow crustal environment, thus giving rise to the Zijinshan alkaline complex^23^.

The Zijinshan Rock Mass is extensively developed, with an outcropping area of about 20 km^2^^23^, Its U content is 0.50–8.88 ppm, and Th content is 1.13–26.30 ppm, which is relatively high, which leads to a high cumulative helium generation amount. Therefore, the Zijinshan Rock Mass has become an important origin of helium within the coal-measure gas in the north of Sanjiao area, and this is also the key factor for the relatively high helium content in the coal-measure gas of the north of Sanjiao area^7^.

### Characteristics of U and Th contents in rocks of different geological ages

This study conducts an analysis of rock samples from outcrops in the field of different geological ages. According to the radioactive decay laws of U and Th, the volume of helium atoms released per gram of rock per year is calculated. Meanwhile, by collecting relevant information such as the stratigraphic thickness and density of rocks of different geological ages, the volume of ^4^He generated by radioactive decay per cubic meter of rock is further calculated (Table 4). The relevant formulas for the decay equations and the half-life (*T*_1/2_) are as follows^24^:

1 $${}_{{92}}^{{238}}U \to {}_{{82}}^{{206}}Pb+8{}_{2}^{4}He+6{}_{0}^{{ - 1}}e,{T_{1/2}}=4.468 \times {10^9}a$$

2 $${}_{{92}}^{{235}}U \to {}_{{82}}^{{207}}Pb+7{}_{2}^{4}He+4{}_{0}^{{ - 1}}e,{T_{1/2}}=7.100 \times {10^8}a$$

3 $${}_{{90}}^{{232}}Th \to {}_{{82}}^{{208}}Pb+6{}_{2}^{4}He+4{}_{0}^{{ - 1}}e,{T_{1/2}}=1.401 \times {10^{10}}a$$

Relying on the radioactive decay mechanisms of U and Th as proposed by Craig^25^ the formula that determines the rate at which ^4^He is generated is:

4 $${\mathrm{J}}\left( {^{4}{\mathrm{He}}} \right)= 1.196 \times 10^{-13} \left[ {\mathrm{U}} \right]+2.897 \times 10^{-14}\left[ {{\mathrm{Th}}} \right]$$

where J(^4^He) is the generation rate of ^4^He, with the unit of cm^3^/(g·a) and represents the volume of ^4^He generated per gram of rock per year, and [U] and [Th] represent the contents of U and Th per gram of rock, with the unit of 10^−6^.

Then the mass of the rock capable of generating helium is:

5 $${M_{rock}}=\rho \times V$$

Where *M*_rock_ represents the mass of the helium source rock, with the unit of grams (g); *ρ* represents the density of the rock, with the unit of grams per cubic centimeter (g/cm^3^); *V* represents the volume of the helium source rock, with the unit of cubic centimeters (cm^3^).

The collected samples were classified according to different geological ages, and the stratigraphic thickness and density of the rocks were investigated. Among them, the maximum thickness of the Shanxi Formation was selected as 120 m; the thickness of the Taiyuan Formation was taken as 60 m; and the thickness of the Benxi Formation of the Carboniferous System was taken as 72 m^26^. The outcrop of the 8th Member of the lower shihezi Formation of the Permian System collected in Fugu has a stratigraphic thickness of 60 m^27,28^. The overall thickness of the Permian System was taken as 240 m, and the thickness of the Benxi Formation of the Carboniferous was taken as 72 m.

The stratigraphic thickness of the Jiehekou Group of the Archaean is 3900 m. In the Lüliang Group of the Archaean, the thickness of the Upper Subgroup of the Upper Archean Lüliang Group exceeds 7300 m, the thickness of the Middle Subgroup exceeds 2040 m, and the thickness of the Lower Subgroup exceeds 6100 m, with a total thickness of 19,340 m and an age of 2.5 Ga^29–32^. The thickness of the Yejishan Group of the Proterozoic is more than 3000 m, and the Lanhe Group of the Proterozoic has a thickness exceeding 2000 m, totaling 5000 m^32^. The thickness of the Cambrian System was taken as 100 m^33^, and the stratigraphic thickness of the Ordovician System is 381 m^34^.

Regarding the density of rocks of different geological ages in the Ordos basin, the density of the upper crust was taken as 2.65 g/cm^3^, the density of the Cambrian was 2.65 g/cm^3^, the density of the Ordovician was 2.70 g/cm^3^, and the density of the Permian-Carboniferous was 2.61 g/cm^3^^4, 35,36^. The average content of the test results was used for the contents of U and Th of the classified samples, and with the help of formula (4), the volume of ^4^He released per gram of rock per year for rocks of different geological ages was calculated. The results show that ^4^He release volume is highest in the Carboniferous and lowest in the Cambrian (Table 4; Fig. 2c). It can be seen that the contents of U and Th play a key role in the volume of ^4^He released by rocks. The volume of ^4^He generated by the radioactive decay of per cubic meter of Archean-Proterozoic rocks during the geological history period is significantly higher than that of other strata (Fig. 2d), indicating that the age of the rocks has a greater influence on the release of helium gas than the contents of U and Th. If you want to accurately calculate the volume of ^4^He generated by the radioactive decay of the rocks in this stratum, it is necessary to clarify the thickness and development area of this stratum. Through investigation, it is found that the development thickness of the Archean-Proterozoic basement is much larger than that of other strata. At the same time, the Archean-Proterozoic basement is widely developed throughout the basin with a large distribution area. Therefore, the volume of ^4^He generated in this stratum is much higher in magnitude than that of other strata.

**Table 4:** The potential of helium gas release from rocks of different geological ages.

System	Age/Ma	U/ppm	Th/ppm	Density/(g/cm^3^)	Area/m	Thickness/m	The volume of ^4^He released per gram of rock per year/m^3^	The volume of ^4^He generated by radioactive decay per cubic meter of rock/10^-3^m^3^
Permian	260	3.14	11.25	2.61	–	240	7.02 × 10^–19^	0.4764
Carboniferous	320	7.21	25.35	2.61	–	72	15.97 × 10^–19^	1.3338
Ordovician	450	1.29	2.74	2.70	–	381	2.34 × 10^–19^	0.2843
Cambrian	500	0.24	0.61	2.65	–	100	0.46 × 10^–19^	0.0610
Proterozoic	1800	3.53	21.93	2.65	–	5000	10.58 × 10^–19^	5.0467
Archean	2500	1.09	5.47	2.65	–	19,340	2.89 × 10^–19^	1.9146

### Characteristics of U and Th contents in different lithologies

The data of 77 samples obtained from the testing and analysis are classified according to lithology, and the volume of helium released per gram of rock per year and the volume of ^4^He generated by radioactive decay per cubic meter of rock for different types of rock samples were calculated. Meanwhile, the thickness, density, and age of rocks with different lithologies were investigated. The research results show that the density of coal in the Ordos basin is taken as 1.42 g/cm^3^ ^26^. The thickness of coal seams in the Carboniferous-Permian exhibits the features of zoning in the east-west direction and partitioning in the north-south direction. Specifically, it is thicker in the western margin (> 10 m), thinner in the central part (< 10 m), and thicker in the eastern part (> 10 m), and it is thicker in the north and thinner in the south. Therefore, the overall thickness of coal in the Carboniferous Benxi Formation and the Permian Shanxi Formation is taken as 25 m^37^. The zircon age of the Taiyuan Formation was determined to be 298.34 ± 0.09 Ma, and this study takes 298 Ma^38^.

The lithology of the Archean-Proterozoic basement is complex, composed of various lithologies such as granite, gneiss, and diorite, etc. The density of the basement rock is taken as 2.85 g/cm^3^^35^, and the age is 2035 Ma^39^. In this study, the Archean samples are from the Jiehekou Group and the Lüliang Group. The thickness of the Jiehekou Group strata is 3900 m. For the Upper Archean of the Lüliang Group, the upper sub-rock group has a thickness exceeding 7300 m, the middle sub-rock group is greater than 2040 m, and the lower sub-rock group is greater than 6100 m. The total thickness amounts to 19,340 m, and the age is taken as 2.5 Ga^29–32^. The Proterozoic samples are collected from the Yejishan Group and the Lanhe Group. The thickness of the Yejishan Group is greater than 3000 m, and that of the Lanhe Group is greater than 2000 m. The overall thickness is taken as 5000 m^40^. Therefore, the total thickness of the basement is taken as 24,340 m.

Bauxite rock primarily consists of hydroxides and oxides. Minerals such as gibbsite, boehmite, and diaspore are commonly present. The density of diaspore minerals ranges from 3.2 to 3.5 g/cm^3^, and the density of bauxite rock is concentrated between 2.6 and 2.85 g/cm^3^. In this study, the density of bauxite rock is taken as 2.65 g/cm^3^^41^, the age is 317 Ma^42^, and the thickness of the bauxite rock in the Benxi Formation is taken as 22.2 m^43^. The density of sandstone in the Benxi Formation is taken as 2.71 g/cm^3^^44^. The total thickness of the sandstone in the Shanxi, Taiyuan and Benxi Formations is taken as 125 m. The lower Shihezi Formation’s 8th member has a thickness of 60 m, and the cumulative thickness reaches 185 m^27,28,45^. The density of mudstone is taken as 2.21 g/cm^3^^12^. In the Upper Paleozoic of the eastern margin of the Ordos basin, the carbonaceous mudstone is relatively thick in the Benxi Formation (with an average thickness of 5.6 m) and the Taiyuan Formation (with an average thickness of 9.2 m), and is relatively thin in the Shanxi Formation, where the average thickness is 3.2 m. The dark mudstone is relatively thin in the Benxi Formation (with an average thickness of 23.8 m) and the Taiyuan Formation (with an average thickness of 27.1 m), and the mudstone in the Shanxi Formation has a large thickness value. On average, it is 43.4 m thick, and the overall total thickness reaches 112.3 m^46^. The density of carbonate rock is 2.77 g/cm^3^^47^. The thickness of carbonate rock in the Majiagou Formation of the Ordovician System is taken as 325.4 m, the thickness of carbonate rock in the Yeli Formation is taken as 44.8 m, and the thickness of carbonate rock in the Taiyuan Formation of the Permian System is taken as 43 m, with a total thickness of 413.2 m, and the age is taken as 450 Ma^26,34^.

For various types of rocks, the contents of U and Th adopt the average values of the test results. With the help of formula (4), the volume of ^4^He released per gram of rock per year for different types of rocks is calculated. The calculation results show that the volume value of ^4^He released by bauxite rock is the highest among all types of rocks (Fig. 3c), while that released by carbonate rock and basement rock is relatively low. In terms of the volume of ^4^He generated by the radioactive decay of rocks per cubic meter, the basement rock and bauxite rock are higher than coal, sandstone, mudstone and carbonate rocks (Table 5; Fig. 3d). However, the development thickness of bauxite rock and coal is relatively thin, and their scales are relatively limited. Basement rock, sandstone and mudstone are widely developed in the study area, and the thickness of the basement is much larger than that of rocks of other lithologies. Therefore, basement rocks significantly contribute to the helium supply in the eastern margin of the Ordos basin.

**Table 5:** The potential of helium release from different lithologies.

Lithology	Formation	Age/Ma	U/ppm	Th/ppm	Density/(g/cm^3^)	Area/m	Thickness/m	The volume of helium atoms released per gram of rock per year/m^3^	The amount of ^4^He produced by radioactive decay per cubic meter of rock/10^-3^m^3^
Basement rock	Pt-Ar	2035	1.93	10.83	2.85	–	24,340	5.53 × 10^− 19^	3.2073
Bauxite rock	C_2_b	317	14.91	50.41	2.65	–	22.2	32.43 × 10^− 19^	2.7243
Coal	C_2_b, P_1_s	298	5.95	21.61	1.42	–	25	13.38 × 10^− 19^	0.5662
Mudstone	C_2_b, P_1_s, P_1_t	298	5.07	16.00	2.21	–	112.3	10.70 × 10^− 19^	0.7047
Sandstone	C_2_b, P_1_s, P_1_t, P_2_h	298	1.75	9.66	2.71	–	185	4.89 × 10^− 19^	0.3949
Carbonate rock	P_1_t, O_1_m, O_1_y, Є_3_f	450	1.75	2.85	2.77	–	413.2	2.95 × 10^− 19^	0.3677

The research in the above 4.2.1 and 4.2.2 indicates that the major factors of effective helium source rocks are not merely limited to the contents of U and Th. In fact, various factors such as the age of the strata (accumulation time of radioactive decay), the thickness of the strata, and the distribution area all play an indispensable and significant role in the process of helium generation, jointly shaping the distribution pattern and enrichment degree of helium resources in the region. These factors also become important reasons why the Ordos basin is more enriched in helium compared with other basins in China.

### The influence of other factors on the generation of helium in helium source rocks

**Fig. 4:**
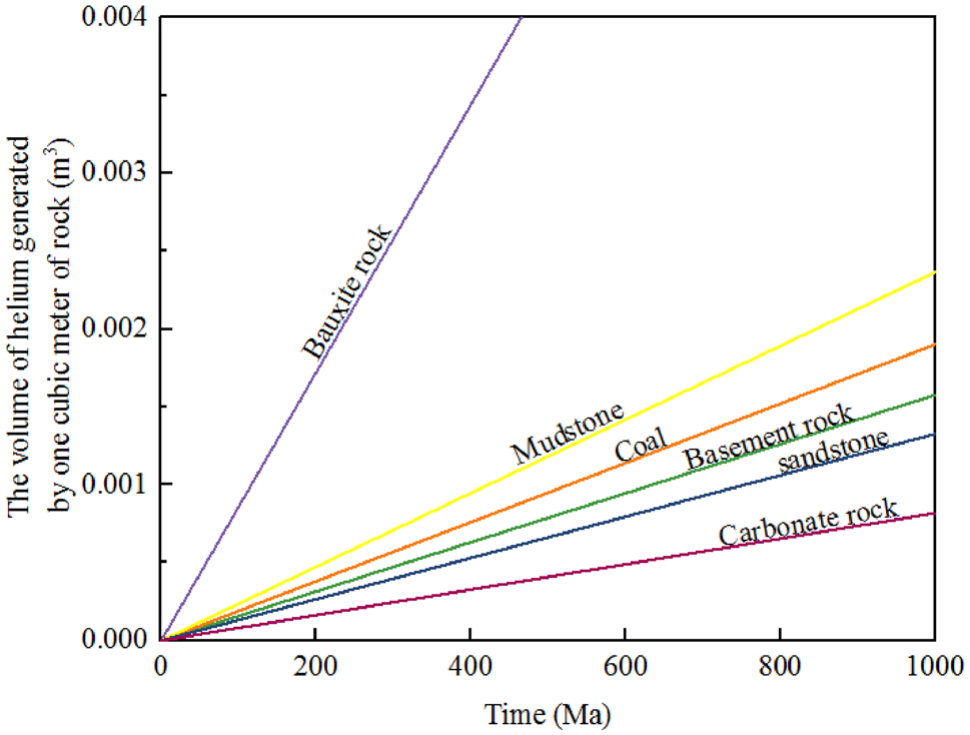
Diagram of the relationship between the amount of Helium generated by per cubic meter of rock and the age of the Rock.

Among various lithologies of rocks, the volume of helium generated by per cubic meter of rock after experiencing the same geological history period is as follows: bauxite rock > mudstone > coal > basement rock > sandstone > carbonate rock(Fig. 4). This is somewhat different from the order of helium generation amounts proposed by Brown in 2010^8^, which is: hot shale > bauxite rock > shale > granite > carbonate rock > sandstone. There are differences in the helium generation rates of different types of rocks in different regions, which are mainly influenced by the elemental composition of the rocks and the environment.

The geological environment in which rocks are located, encompassing several key elements such as tectonic activities and hydrothermal activities, has a significant influence on the amount of helium generated by rocks. In regions with frequent tectonic activities, rocks are often subjected to strong tectonic stress effects such as intense compression and fragmentation, resulting in remarkable changes in their physical structures. Such structural changes not only reshape the decay environment of U and Th elements but also have a profound influence on the migration and preservation conditions of helium within the pore and fracture systems of rocks. For example, a large number of fractures formed by rock fragmentation create more unobstructed channels for the migration of helium, which may accelerate the loss of helium; conversely, the decrease in rock porosity caused by intense compression may impede the migration of helium, causing it to accumulate in local areas.

Hydrothermal activities also play an important role in the process of helium generation by rocks. Hydrothermal activities can not only bring additional heat to rocks, increasing their temperature, but also bring fluids rich in various chemical components. These fluids can promote the dissolution and migration of radioactive elements U and Th, thereby affecting the helium generation rate. In some areas with active hydrothermal activities, there is a strong interaction between hydrothermal fluids and rocks. The chemically active substances in the hydrothermal fluids can destroy the crystal structures of U- and Th-containing minerals, accelerating the decay process of radioactive elements, and ultimately increasing the aggregate quantity of helium produced by rocks.

**Fig. 5:**
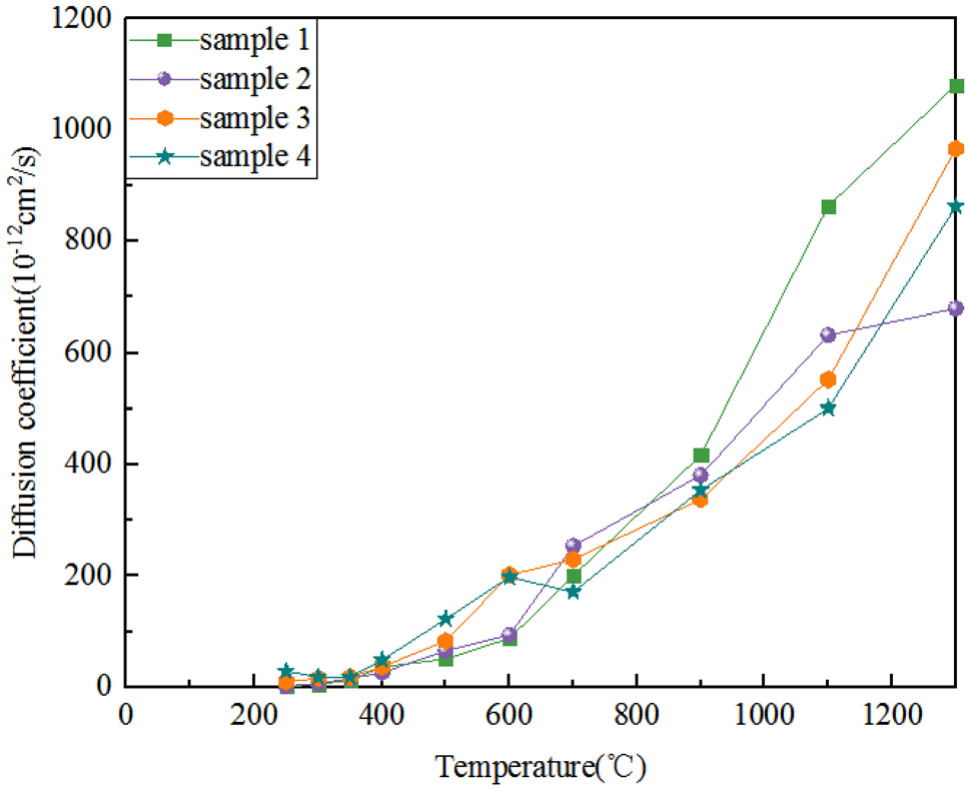
The influence of temperature on the diffusion of helium gas in granite. Data cited from ref.^48^.

Taking the research of Zhang in 2019 as an example^48^, this study delved deeply into the influence mechanism of temperature on the helium release behavior in granite. The research shows that with the increase of temperature, the thermodynamic driving force forHe to escape from the lattice of granite significantly increases, making it easier for ^4^He to be released from granite (Fig. 5). From a macroscopic perspective on the geological time scale, when the temperature is higher than 250 ℃, the crystal structures of minerals containing U and Th undergo irreversible changes due to the high temperature. The adsorption capacity of minerals for helium decreases, resulting in helium being difficult to remain stably in the mineral lattice and being released into the external space of the minerals. It then rapidly migrates to the fluid system in the shallow part of the crust. When ^4^He encounters a suitable gas reservoir during the migration process, it will mix with other gas components in the gas reservoir. Under various physical effects such as buoyancy and capillary force, it enriches and finally forms an accumulation of associated helium and natural gas^49^.

The overall volume of helium produced in the region is comprehensively affected by various factors such as the contents of U and Th, geological age, stratum thickness and volume, and the geological environment. From the perspective of elemental composition, rocks with high concentrations of U and Th serve as the primary sources of helium. Rocks with higher contents of U and Th have a relatively higher helium generation rate. The geological age also plays a crucial role in the helium generation rate. The longer the formation time of rocks, the longer the accumulation time of the decay of radioactive elements like U and Th, and the total amount of helium generation will also increase. For example, the basement rock of the Archean-Proterozoic in the eastern margin of the Ordos basin can have an age of up to 2035 Ma or even older. The long-term decay process enables them to continuously generate helium throughout the long geological history. Although their contents of U and Th are not the highest, due to the extremely long accumulation time of decay, the volume of ^4^He generated per cubic meter of rock through radioactive decay far exceeds that of some other types of rocks. In contrast, for some younger rocks, due to their short formation time and insufficient decay time of radioactive elements, the amount of helium generated is relatively low. The stratum thickness and distribution scale also affect the volume of helium generated. Rocks that are of great thickness and have an extensive distribution area mean that more radioactive elements U and Th participate in the decay reaction. Taking the Benxi Formation of the Carboniferous and the Shanxi Formation of the Permian in the study area as examples, the overall thickness of the coal rocks is taken as 25 m. The relatively small thickness results in a limited amount of radioactive decay, even though the coal may contain a large amount of U and Th elements. Compared with formations with a larger development scale, the volume of helium generated by radioactive decay in this coal layer is small. When calculating the total volume of ^4^He produced by radioactive decay, the volume scale of rocks is a key influencing factor. Rocks with a larger volume scale will have a correspondingly increased total amount of helium generation because they have more sources of U and Th.

### Helium migration channel

**Fig. 6:**
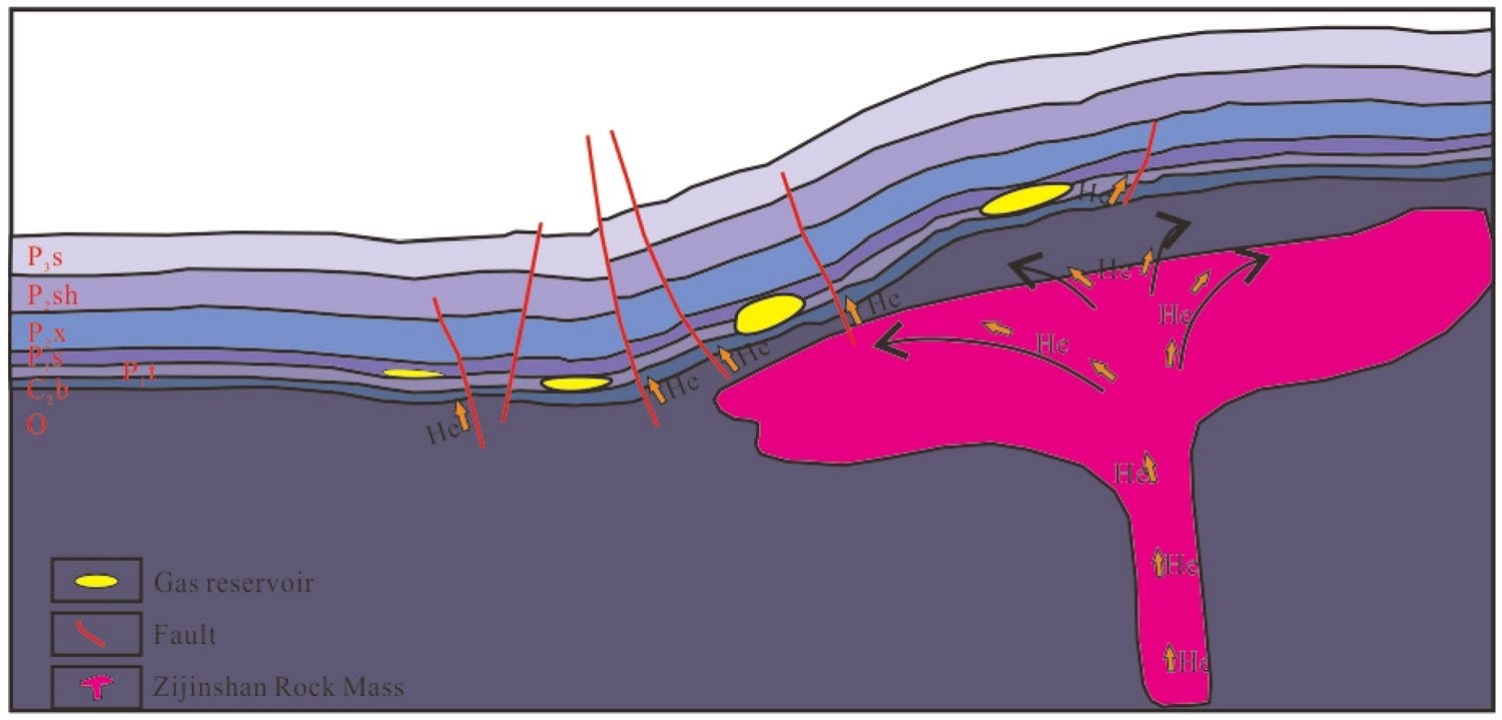
Diagram of the helium accumulation model in the North of Sanjiao Area. Modified from ref.^51^

Coalbed methane is a typical autogenous and self-storage gas reservoir. Its alkane gas is derived from the internal generation of the coal seam and is effectively preserved within the coal seam, which serves as the primary reservoir space. At the same time, the coal seam rich in U and Th elements acts as an important source rock for helium generation, forming an important material basis for the source of helium. However, in this type of gas reservoir, the enrichment of helium mainly depends on the input of exogenous helium^7,24^. Regarding the study area, the coal seams in Shanxi and Taiyuan Formation can provide a small amount of preserved helium sources. The north of Sanjiao area is located above the basement gneiss. A set of thrust faults developed in the Ordovician strata on the western side of the Jinxi flexure zone, and these faults extend upward to the top of the Ordovician but do not penetrate the Carboniferous-Permian coal measures. In addition, the Mesozoic-Cenozoic tectonic uplift of the Lüliang Uplift led to the uplift of the gneiss basement in the eastern margin of the Ordos basin, providing driving force for the upward migration of helium from the basin basement^50^. The Zijinshan Rock Mass developed in the study area provides an extremely important material origin for the helium accumulation in the north of Sanjiao area. This rock mass is closely adjacent to the Carboniferous-Permian reservoirs in space. The helium sources generated by the radioactive decay of U and Th elements rich in their interior, relying on geological structures such as fractures and faults developed around the rock mass as migration channels, are carried by non-hydrocarbon gases such as CH_4_, N_2_, CO_2_ and underground fluid media such as groundwater, and efficiently migrate to the Carboniferous-Permian reservoirs. Then, it gradually enriches in the reservoir spaces such as pores and fractures inside the reservoir, having a significant impact on the helium content in the coalbed methane reservoirs in the study area (Fig. 6).

### Helium reservoir and cap rock conditions

Helium molecules have an extremely small diameter and strong diffusion ability, and they are very likely to disperse in the geological environment. Therefore, the cap rock is crucial for the preservation of helium. The cap rock within the study region is predominantly made up of interbedded mudstone, sandy mudstone, and silty mudstone, and in some local areas, it consists of medium-grained sandstone and fine-grained sandstone^15^. Fine-grained rocks such as mudstone have extremely low permeability due to their small pores and high degree of compaction. They can effectively prevent helium molecules from diffusing through the pores, thus enabling helium to be preserved in the reservoir.

Taking the No. 5 and No. 8 coal seams in the study area as examples, the characteristics of their cap rocks show obvious differences. The roof of the No. 8 coal seam is limestone^52,53^; the direct cap rock of the No. 5 coal seam is mainly mudstone, and the distribution range of the sandstone roof is mainly concentrated in the central area where the coal seam is relatively thin^15^. Limestone and sandstone with developed fractures have poor sealing performance, which will affect the enrichment of helium and directly influence the preservation effect of helium. A cap rock with good sealing ability can ensure that the helium that has migrated to the reservoir will not be easily lost, allowing helium to stably enrich in the reservoir, highlighting the indispensable and important position of the cap rock in the process of helium accumulation.

## Conclusion

1. The test results of coalbed methane samples from 10 wells in the north of Sanjiao area show that the helium content in 8 wells exceeds 0.05%, and the helium content in 4 wells is greater than 0.1%, which has reached the industrial helium extraction standard. This indicates that some areas in the north of Sanjiao area have the potential for developing industrial helium resources.
2. The study on the helium generation rate of rocks in different geological times in the eastern margin of the Ordos basin shows that the helium generation rate presents the characteristics of Carboniferous > Proterozoic > Permian > Archean > Ordovician > Cambrian. In terms of the amount of ^4^He generated by radioactive decay per unit volume of rock: Proterozoic > Archean > Carboniferous > Permian > Ordovician > Cambrian. For rocks of different lithologies, the helium generation rate is bauxite rock > coal > mudstone > basement rock> sandstone > carbonate rock, and the volume of ^4^He produced by radioactive decay per unit volume of rock is basement rock> bauxite rock > mudstone > coal > sandstone > carbonate rock. The results reveal the differences in the helium generation ability and the output of ^4^He of rocks in different geological times and lithologies in this area.
3. The Carboniferous strata and bauxite rock in the study area have high contents of U and Th and a high helium generation rate. Although the helium generation rate of the ancient basement rock (Archean-Proterozoic) is not the highest, the volume of ^4^He generated by radioactive decay per unit volume of rock is the highest among all strata. This indicates that various factors such as the age of the strata (decay accumulation time), the thickness of the strata, and the volume scale affect the total amount of helium generated. Among them, factors including temperature and the geological environment jointly determine the degree to which helium is released from U- and Th-bearing minerals into the external space.
4. Favorable source-reservoir-caprock conditions are the primary factor for helium enrichment. In the north of the Sanjiao area, the developed multi-layered coal seams, bauxite rocks, basement rocks, and the Zijingshan rock mass serve as high-quality helium source rocks. Geological structures such as fractures and faults developed around the Zijingshan rock mass act as migration channels for helium. The mudstone caprock can prevent the helium that has migrated to the reservoir from escaping easily.

## Data Availability

The dataset will be available with the corresponding author based on individual reguests.
